# Knockdown of Regulator of Cullins-1 (ROC1) Expression Induces Bladder Cancer Cell Cycle Arrest at the G2 Phase and Senescence

**DOI:** 10.1371/journal.pone.0062734

**Published:** 2013-05-08

**Authors:** Wei Wang, Zhihong Liu, Ping Qu, Zhengdong Zhou, Yigang Zeng, Jie Fan, Yong Liu, Yifeng Guo, Jianxin Qiu

**Affiliations:** 1 Department of Urology, Shanghai First People’s Hospital, School of Medicine, Shanghai Jiao Tong University, Shanghai, China; 2 Department of Urology, The Fourth Affiliated Hospital of Nantong University (Yancheng First People’s Hospital), Jiangsu, China; University of California Irvine, United States of America

## Abstract

Regulator of Cullins-1 (ROC1) is a key subunit in the Cullin-RING ligase (CRL) protein complex. Overexpression of ROC1 protein is associated with tumor progression and poor prognosis of non-muscle invasive bladder transitional cell carcinoma (NMIBC). This study was designed to assess the effects of ROC1 knockdown in bladder cancer cells and to determine the potential mechanisms involved. A total of 112 bladder cancer tissue specimens were recruited for immunohistochemical analyses of ROC1 overexpression. Bladder cancer cell lines were used to knockdown ROC1 expression using ROC1 siRNA. Our data showed that ROC1 knockdown remarkably inhibited bladder cancer cell growth, arrested cells at the G2 phase of the cell cycle, and induced the p53-dependent cell senescence. Molecularly, G2 arrest was associated with upregulation of p21, p27, cyclin B1, and Cdc2 proteins. ROC1 knockdown induced-senescence functioned through p53/p21 pathway. Knockdown of p21 expression partially rescued ROC1 knockdown-induced growth inhibition in cancer cells. Furthermore, nude mouse xenograft analyses confirmed these *in vitro* data. In conclusion, data from the current study indicate that ROC1 plays an essential role in bladder cancer progression and could serve as a novel anticancer target for bladder transitional cell carcinoma (BTCC).

## Introduction

Bladder cancer is the fourth most common cancer and is the eighth leading cause of cancer deaths among men in the world [1]. Histologically, bladder transitional cell carcinoma (BTCC) is the most common subtype and accounts for ∼90% of all bladder cancers [2]. Clinically, 20% to 30% of newly diagnosed bladder cancer cases have invaded into the muscle layer (called muscle-invasive transitional cell carcinoma, MI-TCC), while an additional 10% to 30% of nonmuscle-invasive bladder cancers will eventually progress to MI-TCC [3]. To date, the treatment of bladder cancer depends on the depth of the tumor invades into the bladder wall; for MI-TCC patients, cisplatin-based chemotherapy is usually recommended following surgery [2]. However, the severe toxicity of chemotherapies and relatively low anticancer efficiency confine its widely application in the clinic and majority of MI-TCC will eventually progress and recurrence, leading to poor prognoses of MI-TCC patients [2], [4]. Therefore, identification of novel effective anticancer targets and agents is of great clinical significance and urgently needed.

Towards this end, we focused on the Cullin-RING ligases (CRL) (also known as Skp1, Cullin, or F-box protein [SCF]), which are the largest family of E3 ubiquitin ligases. Due to the ability to mediate ∼20% ubiquitinated protein substrates for proteasome-targeted degradation [5], CRL play an important role in the ubiquitination of cell cycle-related proteins or other proteins (e.g., DNA replication protein, signal transduction protein, gene transcription factor) [6]. Its dysfunction associates with tumor development and progression, suggesting that CRL could be a potential anticancer target. MLN4924, a small molecule inhibitor to inactivated CRL by inhibiting cullins activity, could effectively inhibit growth of various cancer cells [7], further suggesting the importance of CRL in maintaining tumor growth [8], [9]. However, Regulator of Cullins-1 (ROC1), another key subunit of CRL that heterodimerizes with distinct cullins to constitute the catalytic cores, whose function associated with cancer is poorly understand [5]. ROC1, also known as RING box protein-1 (RBX1), contains a small zinc-binding domain called the RING finger, is an evolutionarily conserved protein from yeast to human and plays an essential role in embryonic development [5]. Aberrant expression of ROC1 leads to CRL dysfunction and causes embryonic lethality [10]. Recently, a few studies have emphasized its role in human cancers since ROC1 may be essential for maintaining genome integrity [11]. In rationale, overexpression of ROC1 caused aberration of protein metabolism due to deregulation of protein post-translational ubiquitylation, and eventually impacting on cancer development and progression. Indeed, ROC1 expression influence the development of skin melanoma by regulating cyclinD1 degradation [12]. Consistently, our preliminary data showed that ROC1 protein is overexpressed in non-muscle-invasive bladder cancer, suggesting its potential role in bladder cancer development and progression. In the present study, we determined the effects of ROC1 knockdown in bladder cancer and the potential underlying mechanisms to provide a novel target for treatment of bladder cancer in future.

## Materials and Methods

### Tissue Specimens

In this study, we first recruited 112 cases BTCC tissue specimens from patients who underwent surgery in Shanghai Jiao Tong University Affiliate First Hospital between January 2004 and May 2006. These patients included 83 males and 29 females and were pathologically diagnosed with primary BTCC and age of these patients was between 30 and 86 years (median age of 64 years). Seventy-one patients underwent transurethral resection, 24 patients underwent partial cystectomy, and 17 patients underwent radical cystectomy. The grade and stage of the tumors were assessed in accordance with the world health organization (WHO) 1973 criteria and American Joint Committee on Cancer (AJCC) 2002 TNM system. Furthermore, distant normal bladder tissues were also collected that were more than 5 cm away from the tumor margin for this study. A protocol for use of human surgical samples was approved by the Medical Ethics Committee of Shanghai First people’s Hospital of Shanghai Jiao Tong University(Permit Number: 2011K047) and written informed consent was obtained from each patient.

### Immunohistochemistry

Archived paraffin-embedded formalin-fixed tissue blocks from these patients were sectioned for 4 µm thick sections and used for immunohistochemistry, and the protocol used was according to Pan’s description [13]. A polyclonal antibody against ROC1 (Abcam, Cambridge, MA) or Ki67 (Epitomics, China) was diluted at 1∶300 and used to immunohistochemically stain these tissue sections using a standard protocol with the Envision Dual Labeled Polymer kit (BioGenex, San Ramon, CA) according to the manufacturer’s instructions. The sections were then counterstained with hematoxylin and independently evaluated by two pathologists.

### Cell Lines and Culture

Human bladder cancer RT4, 5637, BIU87, 253J, T24, and EJ cell lines were purchased from the Chinese Academy of Science (Shanghai, China) and cultured in RPMI 1640 (Gibco, CA, USA) with 10% fetal bovine serum (FBS) (Gibco). Cells were grown in a humidified 5% CO_2_ environment at 37°C. In addition, normal urothelial cells (NUC) were obtained from fresh bladder tissues of 2 young donors (30 and 32 years old), and cultured at 37°C with 5% CO_2_ in keratinocyte serum-free medium (KSFM; Gibco) supplemented with 1% FBS, 100 IU mL^–1^ penicillin, and 100 IU mL^–1^ streptomycin.

### ROC1 siRNA and Transfection

Different siRNA oligonucleotides for various genes (such as ROC1 or p21) were obtained from Invitrogen (Carlsbad, CA) and used for transfection into bladder cancer cells. Briefly, the cells were seeded on 6-well plates overnight and the next day, were transfected with ROC1 siRNA using Lipofectamine 2000 (Invitrogen) according to the manufacturer’s instruction. The ROC1 siRNA sequences were 5′-GACTTTCCCTGCTGTTACCTAA-3′; for p21, 5′-GACCAUGUGGACCUGUCAC-3′; and for the scrambled control, 5′-ACGUGACACGUUCGGAGAA-3′. The cells were then subjected to protein extraction or other assays, as listed below.

### Protein Extraction and Western Blot

Cellular protein was extracted from the cells with or without gene transfection using a lysis buffer. Following quantification, the protein lysates were resolved on a SDS-PAGE gel, transferred onto a PVDF membrane (Millipore, Billerica, MA), and immunoblotted with an antibody against ROC1 (Abcam), GAPDH, Rb, cyclin B1 (Epitomics), p16, p21, p27, p53, pRb, cyclin D1, and cdc2 (Cell Signaling Technology, Danvers, MA) and then with a horseradish peroxidase-conjugated secondary antibody. The bound antibody was visualized using standard chemical luminescence methodology.

### Cell Proliferation and Colony Formation Assays

To assess the altered cell phenotypes by knockdown of ROC1 expression, we transfected cells with siROC1 or siCONT for 24 h and then split and seeded the cells onto 96-well plates with 2,000 cells per well in triplicate. At 24, 48, 72, 96 and 120 h post-transfection, cell proliferation was assessed using the Cell Counting Kit-8 kit (Beyotime, China) according to the manufacturer’s instruction.

For colony formation, the duplicated cells were seeded onto 35-mm culture dishes at 400 cells (253J) or 1000 cells (5637) per well in triplicate. Following 9-day incubation at 37°C, colonies were stained with crystal violet in 50% methanol and the number of colonies of 20 or more cells was counted.

### Flow Cytometric Assay

To detect altered cell cycle distribution, we first transfected siRNA into bladder cancer cells and then fixed them in ice-cold 70% ethanol overnight. The next day, cells were washed twice with ice-cold phosphate buffered saline (PBS) and then stained with propidium iodide (PI; at 20 µg/ml, Sigma) solution for 5 min. The cell samples were then analyzed using a BD FACScan flow cytometer for cell cycle distributions. For mitosis phase assessment, cells were stained with PH3/PI using the Alexa 647-conjugated phospho-histone H3-specific antibody (Cell Signaling Technology) according to the manufacturer’s instruction.

### Cell Senescence Associated-β-Galactosidase Assay

Cell senescence-associated expression of-β-galactosidase (SA-β-Gal) activity was measured using a cell senescence detection kit (Beyotime, China) according to the manufacturer’s instruction. Cells with positive β-galactosidase activity (blue color) at pH 6.0 were counted under a light microscope and averaged.

### Assessment of Cell Morphology and Immunoﬂuorescence Staining of Phospho-γH2AX

Following transfection with siROC1 or siCONT for 24 h, the bladder cancer cells were split and re-seeded into 24-well culture plates and cultured for 72 h. Cell morphology was observed under an inverted microscope. Cells with enlarged and flattened morphological alterations were regarded as senescent-positive. For γH2AX foci staining, the cells were transfected with siROC1 or siCONT for 96 h, and then fixed with 4% paraformaldehyde, permeabilized with 0.5% Triton X for 10 min, and then blocked with 5% bovine serum albumin (BSA). The cells were then incubated with an anti-phospho-γH2AX primary antibody (Epitomics) at 4°C overnight. The cells were further incubated the following day with an Alexa 548-conjugated secondary antibody (Invitrogen, Carlsbad, CA) for 1 h at room temperature, and counterstained with 4, 6-diamidino-2-phenylindole (DAPI) solution (1 µg/mL from Sigma) and reviewed and scored under a fluorescent microscope.

### Quantitative RT-PCR

Total RNA was isolated using Trizol reagent (Invitrogen) and then reversely transcribed to cDNA using a PrimeScript Reverse Transcription System (Takara, China) according to the manufacturer’s instructions. The cDNA samples were then amplified using the SYBR Green master mix kit (Takara). Sequences of the primers used are available upon request. All measurements were performed in triplicate. Amplicon was analyzed using the ΔΔCt method [14].

### *In vivo* Mouse Xenografts Assay

To assess the effects of ROC1 knockdown *in vivo*, we performed a nude mouse(athymic, BALB/C nu/nu) xenograft assay, i.e., 5 × 10^6^ 5637 cells in 50 µl PBS mixed with an equal volume of Matrigel (BD Biosciences) were subcutaneously injected into nude mice (aged 4–6 weeks old). One week following tumor cell inoculation, 12 mice were divided into 2 groups (6 mice in each group, the diameter of the tumor is approximately 0.5 cm) and intratumorally injected with 5 × 10^8^ copies of Lenti-shROC1 in the LT-ROC1 group or 5 × 10^8^ copies of Lenti-shCONT in the LT-CONT, respectively. ShROC1 or control shRNA sequences were according to a previous study [15]. Tumor size was measured every other day using a caliper, and tumor volume was calculated using the equation (L × W^2^)/2 (where L and W represented the longest longitudinal and transverse diameter, respectively). Following 7 weeks observation, the mice were killed and tumor xenografts were removed, weighed and photographed. This study followed animal handling and experimental procedures and approved by the Animal Care and Use Committee of Shanghai First people’s Hospital of Shanghai Jiao Tong University.

### Statistical Analyses

Data were expressed as mean ± standard error of the mean (SEM). Statistical analyses were performed using the Bonferroni t-test method after one-way analysis of variance (ANOVA) for multi-group comparison. Two group comparisons were analyzed using a Student’s t-test. *P*<0.05 was considered statistically significant. All statistical evaluations were performed using SPSS 13.0 (SPSS, Inc., Chicago, IL).

## Results

### Overexpression of ROC1 Protein in BTCC Tissue Specimens and Cell Lines

**Figure 1:**
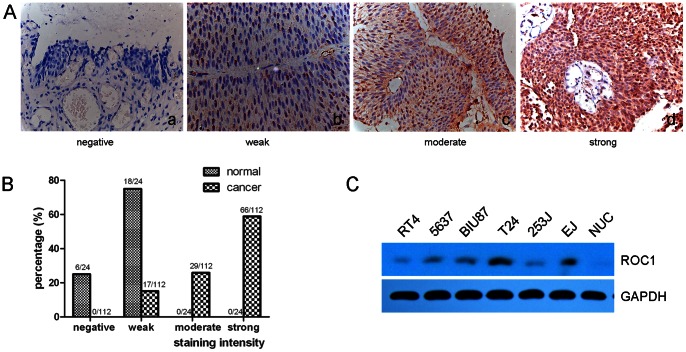
Differential expression of ROC1 protein in bladder cancer tissues and cell lines. A and B, Immunohistochemistry (magnification ×400). Stained tissue microarray sections were scored for four groups according to staining intensity. C, Western blot analyses of ROC1 expression in various BTCC cell lines and normal urothelial cells (NUC).

In this study, we first detected expression of ROC1 protein using immunohistochemistry in 112 cases of normal and BTCC tissue samples, and six cell lines. Our data showed that ROC1 protein was weakly expressed in 18 of 24 (75%) normal bladder urothelium, whereas ROC1 protein was highly expressed in bladder cancer tissues (Fig. 1), i.e., among these 112 BTCC tissue specimens, ROC1 protein was moderately or strongly expressed in 29 (25.9%), and 66 (58.9%) samples, respectively. Moreover, ROC1 protein was expressed in all human BTCC cell lines (RT4, 5637, BIU87, 253J, T24, and EJ) compared to the primary human urothelial cells (Fig. 1).

### Inhibition of BTCC Cell Growth Following Knockdown of ROC1 Expression

**Figure 2:**
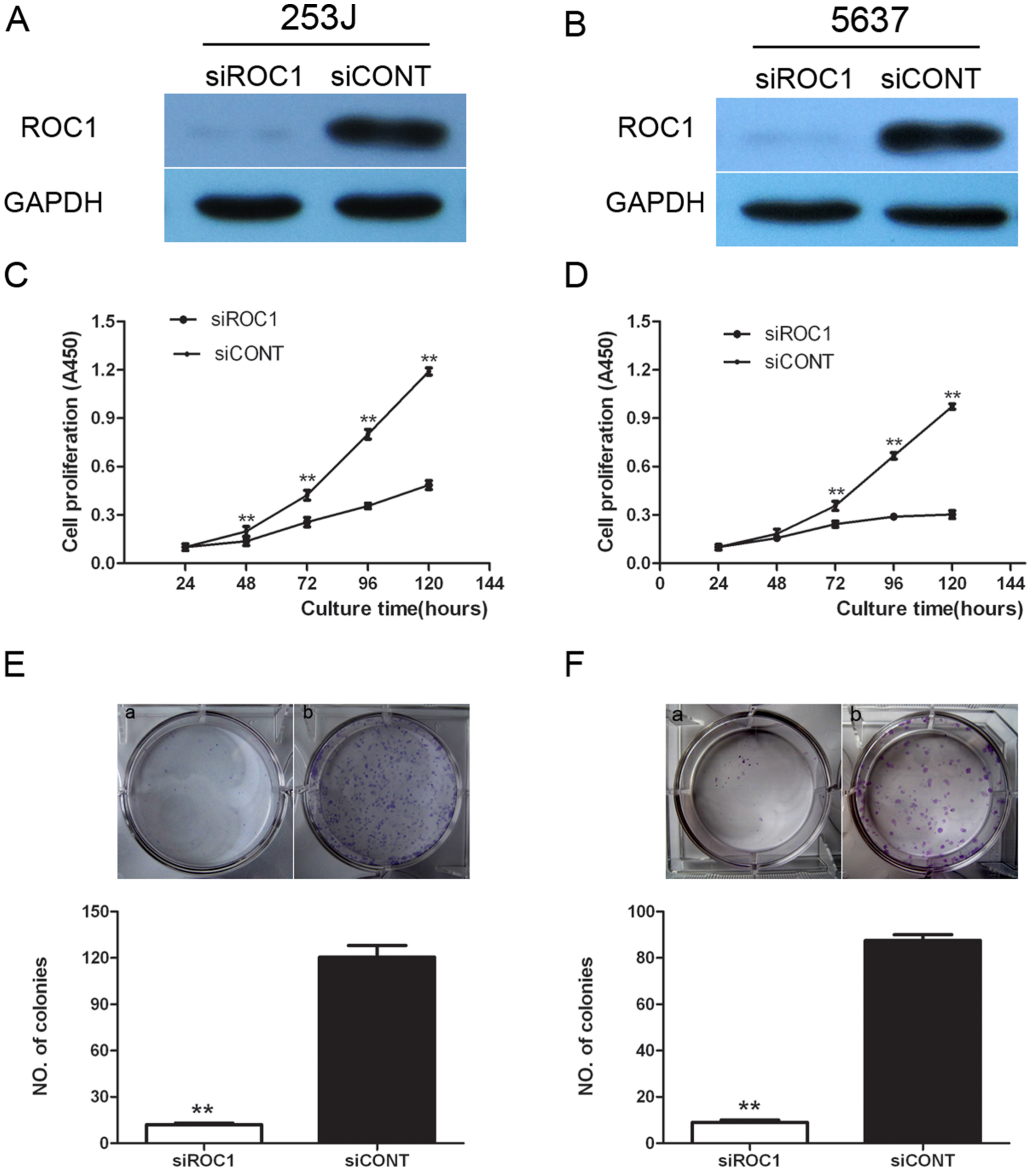
Inhibition of BTCC cell growth following knockdown of ROC1 expression. A, C, E, 253J cells. B, D, F, 5637 cells. These two cell lines were transiently transfected with siROC1 or siCONT for 24–120 h and then subjected to western blot (A and B) analyses of ROC1 expression at 72 h post-transfection, cell viability CCK8 (C and D), or colony formation (E and F) assay. Representative results of three independent experiments are shown as mean ± SEM. **P<0.01.

To assess the role of ROC1 in bladder cancer, we knocked down ROC1 expression in BTCC cell lines using ROC1 siRNA and a scrambled siRNA as control. Our data showed that ROC1 siRNA successfully knocked down expression of ROC1 protein in 253J and 5637 cells (Fig 2A and B).

Subsequently, we evaluated the effects of siROC1 knockdown in these bladder cancer cells and found that siROC1 cells grew remarkably slower than those of the siCONT cells (*P*<0.01 at 72 h, 96 h, and 120 h; Fig. 2C and D). Colony formation assay further showed that the number of colonies was reduced to 5- to 10-fold in siROC1 cells compared to that of siCONT cells (*P*<0.01; Fig. 2E and F).

### ROC1 Knockdown Arrested Cells at the G2 Phase of the Cell Cycle

**Figure 3:**
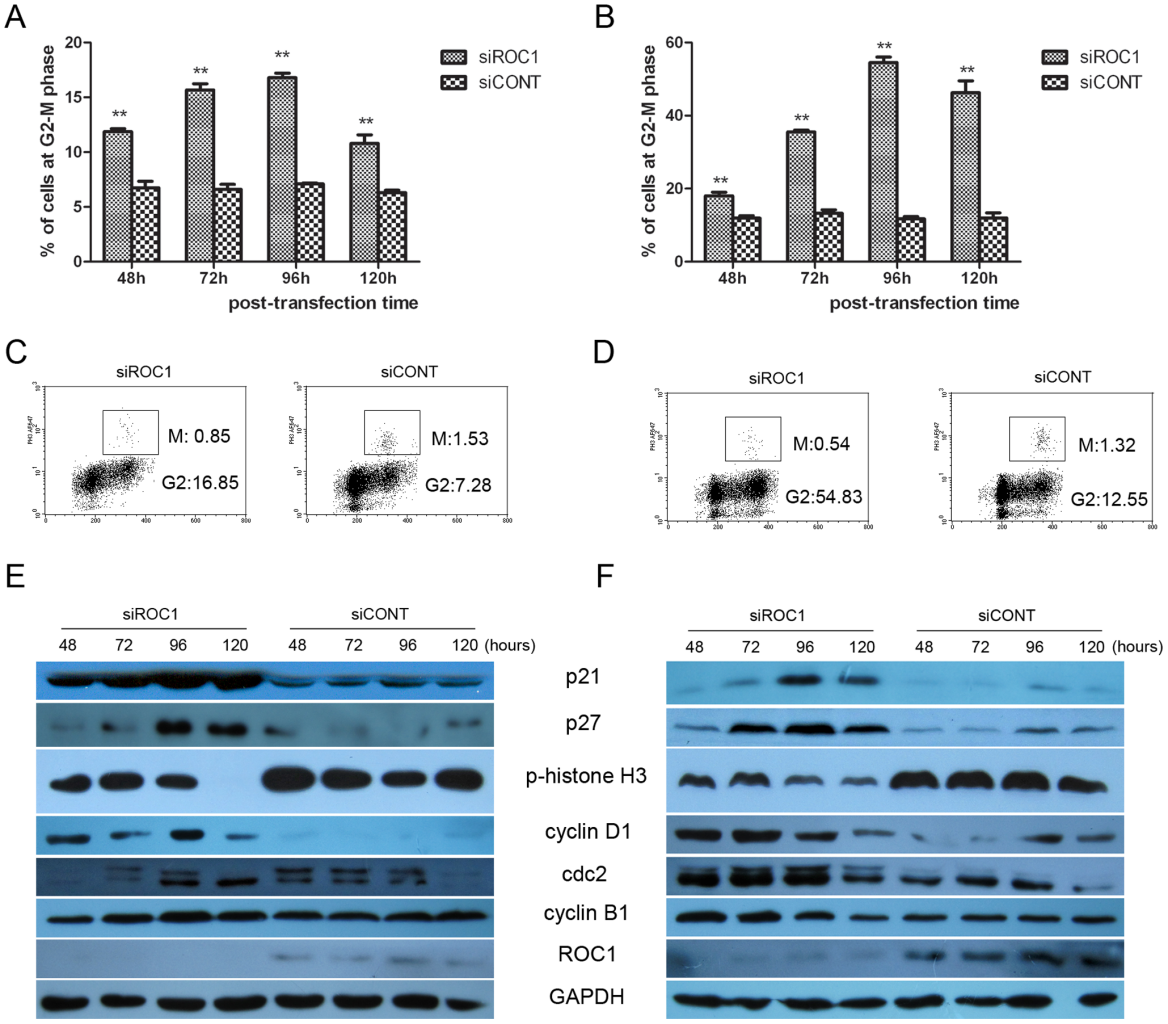
Induction of BTCC cell arrest at cell cycle G2-M phase following ROC1 knockdown. A and B, ROC1 knockdown induced G2-M cell cycle arrest in 253J (A) and 5637 (B) cells. The cells were transfected with siROC1 or siCONT for 48–120h, and cell cycle profile was detected using FACS analyses following Propidium Iodide (PI) staining. C and D, ROC1 knockdown induced G2 cell cycle arrest. 253J (C) and 5637 (D) cells were transfected with siROC1 or siCONT for 96h, and subjected to FACS analysis after phospho-histone 3(PH3)/PI double staining. E and F, Expression of cell cycle-associated protein in 253J (E) and 5637 (F) cells were examined using western blot post-transfection at the indicated time interval. Representative results of three independent experiments are shown. Columns, mean of three independent experiments; bars, SEM. ** *P*<0.01.

We determined cell cycle distribution following ROC1 knockdown in bladder cancer cells and found that both 253J and 5637 cells after ROC1 knockdown arrested at the G2-M phase of cell cycle. Specifically, 253J cells following transfected with ROC1 siRNA for 48, 72, 96, 120h increased the G2-M phase to 11.88±0.27%, 15.68±0.56%, 16.8±0.42%,and 10.8±0.78%, respectively, compared to 6 to 8% in the control cells (Fig 3A), while 5637 cells were to 18±1.41%, 35.5±0.73%, 54.5±2.12% and 46.27±4.62%, respectively, compared to 11 to 14% in the control cells (Fig 3B). These data demonstrate that G2-M arrest in both cells reached peak at 96h post-transfection.

We then investigated expression of the G2-M transition-related regulators, i.e., the G2-M transition of the cell cycle is regulated by a complex of cdc2 and a B-type cyclin. Indeed, our data showed that Cdc2 and cyclin B1 expressions were substantially up-regulated following ROC1 knockdown in both 253J (Fig. 3E) and 5637 cells (Fig. 3F). We also determined the expression of CDK inhibitors, such as p21 and p27 and found that p21 and p27 were significantly accumulated upon ROC1 knockdown in both cell lines (Fig 3E and F). Cyclin D1, a well-known cell cycle regulator of G1 phase, was also markedly induced by ROC1 knockdown in both cell lines (Fig. 3E&F).

To further examine ROC1 knockdown arrested cancer cells in the G2 or M phases, we performed FACS analyses following phospho-histone H3 (PH3)/propidium iodide (PI) double staining of these cell lines. Histone H3 is an indicator of mitosis when phosphorylated at Ser10 during the M phase, but not at the G2 phase [16]. Compared with siCONT cells, siROC1 showed less phosphor-histone H3-positive staining in both 253J and 5637 cells (Fig 3C and D), indicating that siROC1 arrested cells in the G2 phase rather than the M phase. Consistent with these results, western blot data showed that ROC1 knockdown reduced PH3 expression (Fig. 3E&F). Decreased expression of PH3 protein and reduced mitotic cells indicate that ROC1 knockdown arrested cells at the G2 phase, but prevented cells from entering the M-phase.

### ROC1 Knockdown Induced Bladder Cancer 253J Cell Senescence Through the p53/p21 Pathway

**Figure 4:**
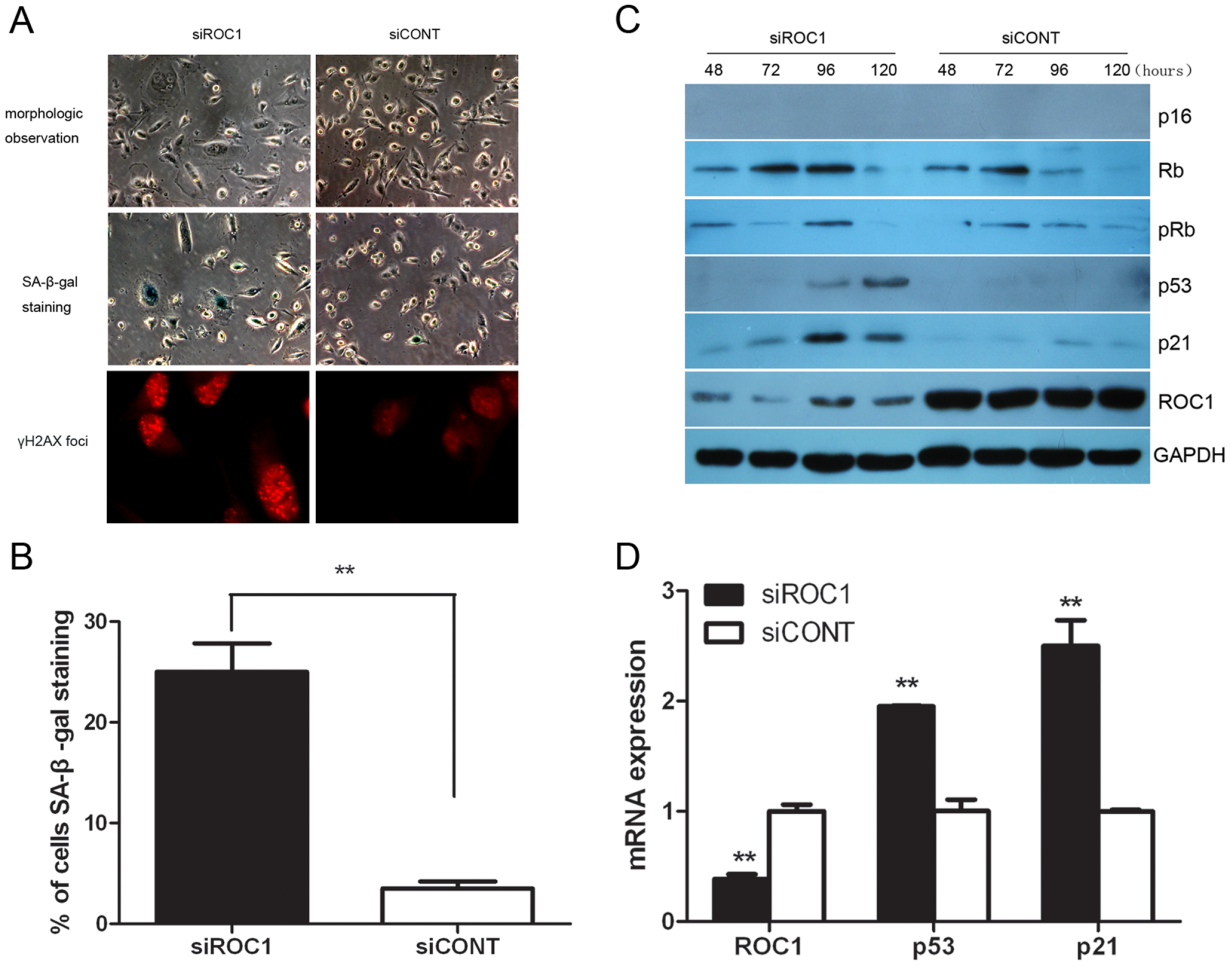
ROC1 knockdown-induced cellular senescence in 253J cells through p53/p21 pathway. A and B, 253J cells were transfected with siROC1 or siCONT for 96h, followed by morphologic observation (A, top panels, magnification ×400), SA-β-gal staining (A, middle panels, magnification ×400) and quantified with positively stained cells (B) and immunoﬂuorescence staining of phospho-γH2AX (A, bottom panels, magnification ×400). C, Expression of senescence-associated pathway protein in 253J cells was examined using western blot post-transfection at the indicated time intervals. D, ROC1, p53, p21 mRNA in 253J cells at 72h post-transfection was examined using quantitative real-time PCR. Representative results of three independent experiments are shown. Columns, mean of three independent experiments; bars, SEM. ** *P*<0.01.

Morphologically, ROC1 knocked down 253J cells became enlarged and flattened, morphology commonly observed in senescent cells (Fig. 4A, top panels), but did not appeared in 5637 cells. We further determined senescence-associated β-galactosidase (SA-β-gal) activity (Fig. 4A, middle panels), a specific marker of senescent cells. As shown in Fig. 4B, approximately 25% of 253J cells were stained positively following 96h ROC1 siRNA transfection, compared to less than 4% positive staining in the control cells (*P*<0.01). In addition, we assessed phospho-γH2AX expression using immunoﬂuorescence staining, which is a marker for cell senescence. Our data showed that 253J cells transfected with siROC1 had more γH2AX foci compared to that of the siCONT cells (Fig. 4A, bottom panels), further indicating that ROC1 knockdown induced 253J cells senescence.

The p16/RB and p53/p21 axes are two major senescence-associated pathways in response to various stressors. Since p16 gene is homozygousely deleted in 253J cells [17], p16 protein was undetectable in 253J cells before and after ROC1 siRNA transfection (Fig. 4C). However, both total and phosphorylated Rb protein levels were not accumulated upon ROC1 knockdown (Fig. 4C), indicating that ROC1 knockdown-induced 253J cell senescence is independent of the p16/RB gene pathway. Our data showed significant upregulation of p53 and p21 proteins in siROC1 cells at 96h and 120h following ROC1 siRNA transfection (Fig. 4C), and *p53* together with *p21* mRNA upregulation were also observed using quantitative real-time PCR (Fig. 4D). Our current data indicated that the p53/p21 gene pathway mediated the effects of ROC1 knockdown on bladder cancer cell senescence, since 253J cells do not express p16, but have a wild type of p53, whereas 5637 cells expressed mutated p53.

### Effects of ROC1 Knockdown on Inhibition of Tumor Cell Growth Through p21 Expression

**Figure 5:**
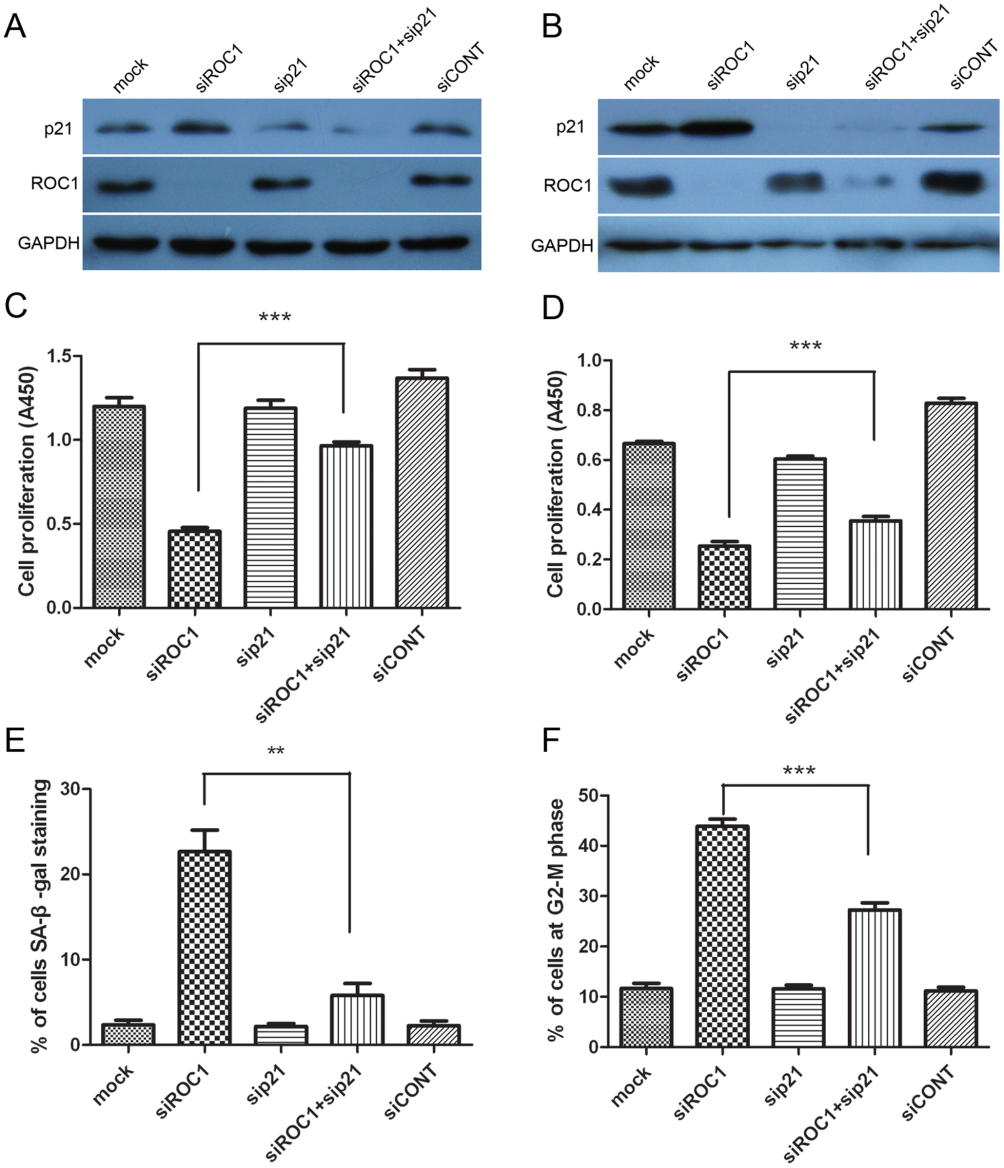
ROC1 knockdown-induced bladder cancer cell growth inhibition was partially rescued by suppression of p21 expression. 253J and 5637 cells were transfected with the indicated siRNA for 96h, and subjected to western blot analyses (A and B), CCK8 assay (C and D), senescence analyses with SA-β-gal staining (E), and FACS analysis with PI staining (F). Representative results of three independent experiments are shown. Columns, mean of three independent experiments; bars, SEM. ** *P*<0.01 and *** *P*<0.001.

To consider whether p21 is accumulated in both G2 arrest and senescence induced by ROC1 knockdown, we further determined the role of p21 in mediating the effects of ROC1 siRNA on regulation of bladder cancer cell growth. Our data showed that simultaneous abrogation of p21 and ROC1 using siRNAs markedly attenuated p21 protein expression (Fig. 5A&B) and attenuated cell growth inhibition caused by ROC1 knockdown in both 253J and 5637 cells (Fig. 5C&D). In 253J cells, SA-β-Gal staining showed that knockdown of p21 expression decreased the rate of ROC1 siRNA-induced cell senescence (Fig. 5E), whereas in 5637 cells, ROC1 silencing induced G2-M arrest was markedly decreased by p21 knockdown (Fig. 5F).

### Effects of ROC1 siRNA on Regulation of 5637 Cell Xenografts Formation and Growth

**Figure 6:**
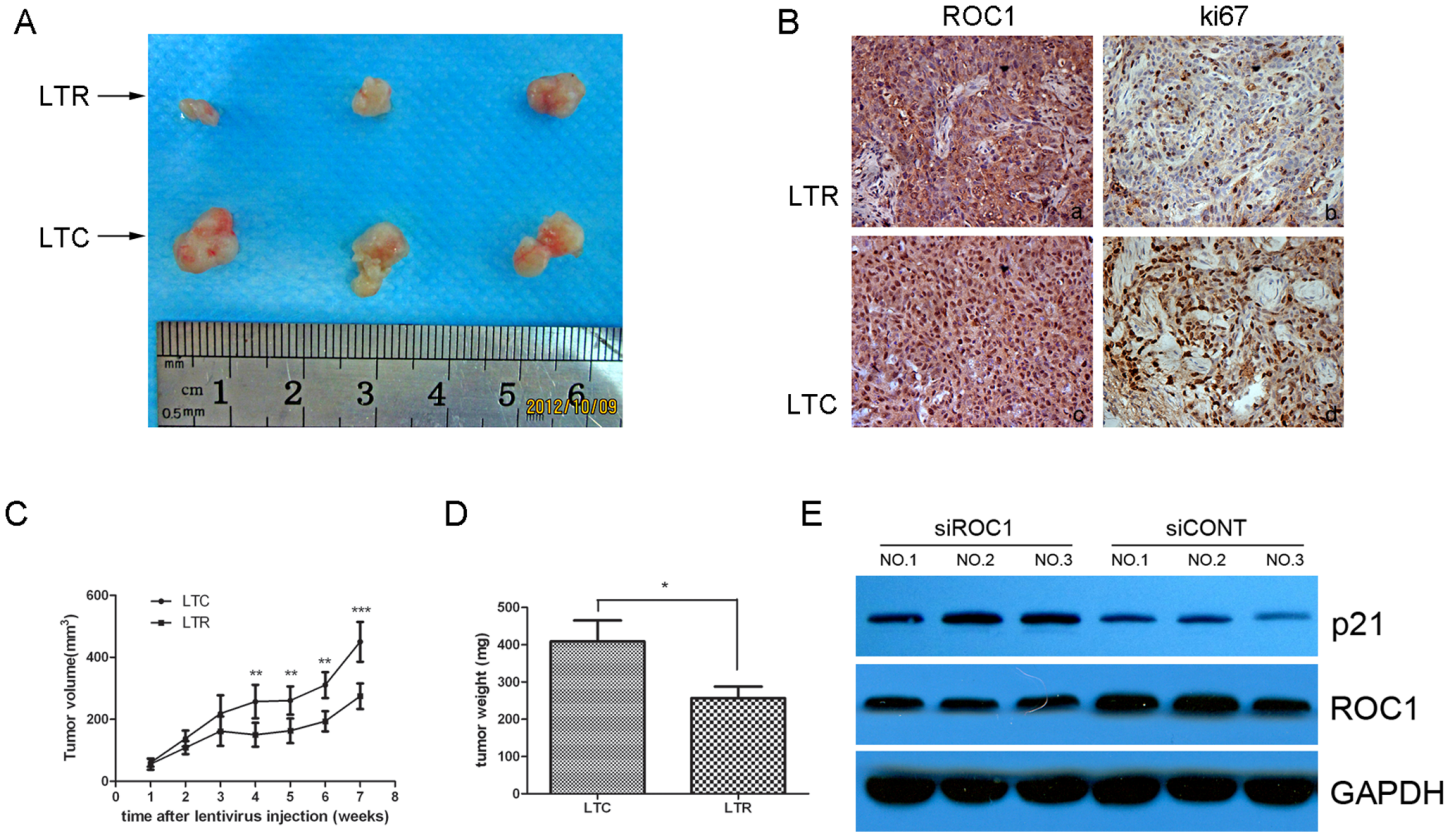
ROC1 knockdown inhibited the growth of bladder cancer cell 5637 xenografts *in vivo.* A, Representative photographs of tumors isolated from nude mice in each group 7 weeks following injection of LTR and LTC. B, IHC staining of xenografts tissues with the indicated antibody in both groups. C and D, Tumor growth curve (C) and tumor weight (D) in both groups. E, ROC1 and p21 protein in extracted protein from xenografts in both groups were determined using western blot analyses. At the end of the experiments, tumor tissues were excised from each mouse and weighed. Columns, mean weight of 5 tumors from individual mice in each group; bars, SEM. * *P*<0.05, ** *P*<0.01 and *** *P*<0.001.

We have showed *in vitro* effects of ROC1 knockdown in bladder cancer cell lines. We sought to confirm these data in nude mouse xenografts. As shown in Fig. 6A&C, intratumoral injection of LT-ROC1 resulted in a significant decrease in growth rate of tumors compared with the control group treated with LT-CONT (*p*<0.01). Immunohistochemistry staining of Ki67 indicated that LT-ROC1 injection reduced nude mice xenografts proliferation (Fig. 6B). The tumor weight in the LT-ROC1 xenografts was significantly lower than that of the LT-CONT group (*p*<0.05). Western blot data showed that p21 protein was upregulated in the LT-ROC1 injection group (Fig. 6E).

## Discussion

In the present study, we reported that ROC1 protein was overexpressed in 112 bladder cancer tissue specimens and 6 cell lines compared to normal tissues and cells. Knockdown of ROC1 expression inhibited bladder cell growth and induced G2 cell cycle arrest and p53-dependent cell senescence. Molecularly, knockdown of ROC1 expression upregulated expression of p21, p27, cyclin B1 and Cdc2 proteins. ROC1 knockdown-induced 253J cells senescence was mediated through the p53/p21 gene pathway, and knockdown of p21 expression partially rescued ROC1 knockdown-induced cancer growth inhibition in BTCC cells. Our nude mouse xenograft analyses further confirmed our *in vitro* data. Current data suggest that ROC1 plays an important role in bladder cancer progression, and targeting ROC1 protein is a potential anticancer strategy for bladder cancer.

Previous studies showed ROC1 overexpression in various cancers and associated with tumor progression [15], [18]. In the present study, we confirmed these data in bladder cancer tissues and cell lines. We further assessed the role of ROC1 protein in bladder cancer by knockdown of ROC1 protein expression in two different bladder cancer cell lines. We showed that ROC1 knockdown potently inhibited bladder cancer cell growth *in vitro* and *in vivo*. Mechanistically, ROC1 knockdown induced cell cycle G2 phase arrest and cell senescence in bladder cancer cell lines.

Cell cycle G2-M phase is a common cell cycle checkpoint mechanism in response to external stresses and induction of G2-M arrest is a useful strategy in treating various human cancers [19]. Molecularly, Cdc2 kinase and cyclin B1 constitute M-phase promoting factor (MPF) that control G2-M transition and M phase progression [20], kinase activity of which is negatively regulated by CDK-inhibitors (CDKIs, such as p21, p27) and Cdc2 inhibitory kinases (such as WEE1) [21]. In our current study, we found that ROC1 knockdown remarkably induced G2-M arrest by upregulating expression of cyclin B1/Cdc2 protein in both 5637 and 253J cells. Moreover, our additional data indicated that ROC1 knockdown arrested bladder cancer cells at G2 phase, although the defined mechanisms are unclear. The conceivable explanation may be that ROC1 knockdown induced CRL inactivation that caused accumulation of some special CRL substrates (such as p21, p27 and WEE1). Our current data showed that ROC1 knockdown induced expression of p21 and p27. In addition, cyclin D1, another well-known CRL substrate [22], was also markedly induced by ROC1 knockdown in both cell lines. However, we did not observe significant changes in cyclin D1-associated G1-S phase arrest, which may be since ROC1 knockdown arrested cells at G2-phase cells are prevented from entering into the G0–G1 phase.

Furthermore, we also revealed that ROC1 knockdown induced p53 wild type bladder cancer 253J cells into senescence through a p53-dependent way. Induction of cellular senescence was regarded as an important tumor suppressive mechanism [23], [24]. Various stresses (such as telomere dysfunction, oncogene activation, DNA damage, and other stimuli) can all initiate cellular senescence [23]. Senescence is mainly mediated by two tumor suppressor pathways: p53/p21 and p16/pRB [23], [24]. The current study showed that ROC1 knockdown induced significant cellular senescence in p53 wild-type 253J cells, but did not induce p53 mutated 5637 cells. Furthermore, blockade of the p53/p21 pathway by knockdown of p21 expression significantly alleviated ROC1 knockdown-induced senescence. These findings suggest that ROC1 knockdown-induced senescence is associated with a functional p53/p21 pathway. However, Jia et al. showed that ROC1 knockdown-induced senescence of lung and cervical cancer cell lines in a p53- or pRB- independent manner [15]. Another similar question is what determines cells to undergo cell cycle arrest or senescence or other cell death types. The exact mechanism remains unknown, and we suppose that this choice may be associated with cell line dependence (such as *p*53 gene status, specificity of CRL substrates, duration of stress, et.al) and intensity of external stress [25] (such as DNA damage response, oxidative stress, et.al). Thus, further study is needed to clarify the defined mechanisms responsible for ROC1 knockdown-induced bladder cancer cell death.

Additionally, we also observed that ROC1 knockdown inhibits tumor growth of 5637 cells in *in vivo* nude mouse xenograft with similar mechanisms. Different stages of bladder cancer showed differential therapeutic responsiveness to radiotherapy and chemotherapy [26]. One determinant factor of therapeutic susceptibilities is *TP53* gene status [27]. Cells with dysfunctional p53 protein showed increased resistance to radiotherapy or chemotherapy due to reduced DNA damage response [3], [26]. The present study demonstrated that ROC1 knockdown inhibited bladder cancer cell growth regardless of p53 status. This result could have an important impact for the treatment of bladder cancer. ROC1 knockdown using gene interference or pharmacological inhibition could be a novel approach to strengthen responsiveness of radiotherapy and chemotherapy of bladder cancer patients. So, further study will investigate such approaches in effectively control bladder cancer.

In conclusion, the present study showed that ROC1 plays an important role in BTCC progression, and ROC1 could be a novel anti-cancer target in BTCC. Further studies clarifying the detailed mechanisms of ROC1 involvement in BTCC are needed to validate our primary results.

## References

[1] Siegel R, Naishadham D, Jemal A (2012) Cancer statistics, 2012. CA Cancer J Clin 62: 10–29. 22237781 10.3322/caac.20138

[2] Kaufman DS, Shipley WU, Feldman AS (2009) Bladder cancer. Lancet 374: 239–249. 19520422 10.1016/S0140-6736(09)60491-8

[3] Mitra AP, Cote RJ (2009) Molecular Pathogenesis and Diagnostics of Bladder Cancer. Annual Review of Pathology: Mechanisms of Disease 4: 251–285. 10.1146/annurev.pathol.4.110807.092230 18840072

[4] Jacobs BL, Lee CT, Montie JE (2010) Bladder Cancer in 2010: How Far have We Come? CA Cancer J Clin 60: 244–272. 20566675 10.3322/caac.20077

[5] Petroski MD, Deshaies RJ (2005) Function and regulation of cullin–RING ubiquitin ligases. Nature Reviews Molecular Cell Biology 6: 9–20. 15688063 10.1038/nrm1547

[6] Nakayama KI, Nakayama K (2006) Ubiquitin ligases: cell-cycle control and cancer. Nature Reviews Cancer 6: 369–381. 16633365 10.1038/nrc1881

[7] Soucy TA, Smith PG, Milhollen MA, Berger AJ, Gavin JM, et al (2009) An inhibitor of NEDD8-activating enzyme as a new approach to treat cancer. Nature 458: 732–736. 19360080 10.1038/nature07884

[8] Milhollen MA, Traore T, Adams-Duffy J, Thomas MP, Berger AJ, et al (2010) MLN4924, a NEDD8-activating enzyme inhibitor, is active in diffuse large B-cell lymphoma models: rationale for treatment of NF-{kappa}B-dependent lymphoma. Blood 116: 1515–1523. 20525923 10.1182/blood-2010-03-272567

[9] Yang D, Tan M, Wang G, Sun Y (2012) The p21-dependent radiosensitization of human breast cancer cells by MLN4924, an investigational inhibitor of NEDD8 activating enzyme. PLoS ONE 7: e34079. 22457814 10.1371/journal.pone.0034079 PMC3310880

[10] Tan M, Davis SW, Saunders TL, Zhu Y, Y S (2009) RBX1/ROC1 disruption results in early embryonic lethality due to proliferation failure, partially rescued by simultaneous loss of p27. Proc Natl Acad Sci 106: 6203–6208. 19325126 10.1073/pnas.0812425106 PMC2669381

[11] Jia L, Bickel JS, Wu J, Morgan MA, Li H, et al (2011) RBX1 (RING box protein 1) E3 ubiquitin ligase is required for genomic integrity by modulating DNA replication licensing proteins. J Biol Chem 286: 3379–3386. 21115485 10.1074/jbc.M110.188425 PMC3030344

[12] Nai G, Marques M (2011) Role of ROC1 protein in the control of cyclin D1 protein expression in skin melanomas. Pathology - Research and Practice 207: 174–181. 10.1016/j.prp.2011.01.001 21300445

[13] Pan H, Hanada S, Zhao J, Mao L, Ma MZ (2012) Protein secretion is required for pregnancy-associated plasma protein-A to promote lung cancer growth in vivo. PLoS ONE 7: e48799. 23152806 10.1371/journal.pone.0048799 PMC3494721

[14] Livak KJ, Schmittgen TD (2001) Analysis of Relative Gene Expression Data Using Real-Time Quantitative PCR and the 2−ΔΔCT Method. Methods 25: 402–408. 11846609 10.1006/meth.2001.1262

[15] Jia L, Soengas MS, Sun Y (2009) ROC1/RBX1 E3 Ubiquitin Ligase Silencing Suppresses Tumor Cell Growth via Sequential Induction of G2-M Arrest, Apoptosis, and Senescence. Cancer Research 69: 4974–4982. 19509229 10.1158/0008-5472.CAN-08-4671 PMC2744327

[16] Xu B, Kim S-t, Kastan MB (2001) Involvement of Brca1 in S-Phase and G2-Phase Checkpoints after Ionizing Irradiation. Molecular and Cellular Biology 21: 3445–3450. 11313470 10.1128/MCB.21.10.3445-3450.2001 PMC100266

[17] Lee CT, Seol JY, Park KH, Yoo CG, Kim YW, et al (2001) Differential effects of adenovirus-p16 on bladder cancer cell lines can be overcome by the addition of butyrate. Clin Cancer Res 7: 210–214. 11205911

[18] Yang D, Li L, Liu H, Wu L, Luo Z, et al (2013) Induction of autophagy and senescence by knockdown of ROC1 E3 ubiquitin ligase to suppress the growth of liver cancer cells. Cell Death Differ 20: 235–247. 22935614 10.1038/cdd.2012.113 PMC3554346

[19] Gabrielli B, Brooks K, Pavey S (2012) Defective cell cycle checkpoints as targets for anti-cancer therapies. Front Pharmacol 3: 9. 22347187 10.3389/fphar.2012.00009 PMC3270485

[20] Malumbres M, Barbacid M (2005) Mammalian cyclin-dependent kinases. Trends Biochem Sci 30: 630–641. 16236519 10.1016/j.tibs.2005.09.005

[21] Malumbres M, Barbacid M (2009) Cell cycle, CDKs and cancer: a changing paradigm. Nature Reviews Cancer 9: 153–166. 19238148 10.1038/nrc2602

[22] Lin DI, Barbash O, Kumar KG, Weber JD, Harper JW, et al (2006) Phosphorylation-dependent ubiquitination of cyclin D1 by the SCF(FBX4-alphaB crystallin) complex. Mol Cell 24: 355–366. 17081987 10.1016/j.molcel.2006.09.007 PMC1702390

[23] Campisi J, d’Adda di Fagagna F (2007) Cellular senescence: when bad things happen to good cells. Nature Reviews Molecular Cell Biology 8: 729–740. 17667954 10.1038/nrm2233

[24] Campisi J (2005) Senescent Cells, Tumor Suppression, and Organismal Aging: Good Citizens, Bad Neighbors. Cell 120: 513–522. 15734683 10.1016/j.cell.2005.02.003

[25] Schauble S, Klement K, Marthandan S, Munch S, Heiland I, et al (2012) Quantitative model of cell cycle arrest and cellular senescence in primary human fibroblasts. PLoS ONE 7: e42150. 22879912 10.1371/journal.pone.0042150 PMC3413708

[26] Prasad SM, Decastro GJ, Steinberg GD (2011) Urothelial carcinoma of the bladder: definition, treatment and future efforts. Nat Rev Urol 8: 631–642. 21989305 10.1038/nrurol.2011.144

[27] Mitra AP, Birkhahn M, Cote RJ (2007) p53 and retinoblastoma pathways in bladder cancer. World Journal of Urology 25: 563–571. 17710407 10.1007/s00345-007-0197-0

